# Nucleotide Diversity and Selection Signature in the Domesticated Silkworm, *Bombyx mori*, and Wild Silkworm, *Bombyx mandarina*

**DOI:** 10.1673/031.011.15501

**Published:** 2011-11-14

**Authors:** Yi Guo, Yi-Hong Shen, Wei Sun, Hirohisa Kishino, Zhong-Huai Xiang, Ze Zhang

**Affiliations:** ^1^ The Key Sericultural Laboratory of Agricultural Ministry, Southwest University, Chongqing 400715, China; ^2^ The Laboratory of Biometrics and Bioinformatics, Graduate School of Agriculture and Life Science, The University of Tokyo, 1-1-1, Yayoi, Bunkyo, Tokyo, 113-8657, Japan; ^3^ The Institute of Agricultural and Life Sciences, Chongqing University, Chongqing 400044, China

**Keywords:** bottleneck, domestication, nucleotide diversity, selection

## Abstract

To investigate the patterns of nucleotide diversity in domesticated silkworm, *Bombyx mori* L. (Lepidoptera: Bombycidae) and its wild relative, Chinese wild silkworm, *Bombyx mandarina* Moore, we sequenced nine nuclear genes. Neutrality test and coalescent simulation for these genes were performed to look at bottleneck intensity and selection signature; linkage disequilibrium (LD) within and between loci was employed to investigate allele association. As a result, *B. mori* lost 33–49% of nucleotide diversity relative to wild silkworm, which is similar to the loss levels found in major cultivated crops. Diversity of *B. mori* is significantly lower than that of *B. mandarina* measured as *π*_total_ (0.01166 vs. 0.1741) or *θ*_W_(0.01124 vs. 0.02206). Bottleneck intensity of domesticated silkworm is 1.5 (in terms of *k* = *N_b_*/*d*, *N_b_*-bottleneck population size; *d*-bottleneck duration) with different durations. Gene *DefA* showed signature of artificial selection by all analysis methods and might experience strong artificial selection in *B. mori* during domestication. For nine loci, both curves of LD decay rapidly within 200 bp and drop slowly when distance is > 200 bp, although that of *B. mori* decays slower than *B. mandarina* at loci investigated. However, LD could not be estimated at *DefA* in *B. mori* and at *ER* in both silkworms. Elevated LD observed in *B. mori* may be indicator of selection and demographic events.

## Introduction

The typical outcome of domestication of crop plants and animals is a genome-wide loss of genetic diversity (Tanksley and McCouch 1997). Although many forces can shape patterns of genetic diversity and structure in crops and domestic animals, one of the most influential factors is the population bottleneck that occurs during the founding of a new domestic lineage (Eyre-Walker et al. 1998). In addition, natural or artificial selection, selective sweep, population extension and subdivision can often change allele frequency and distribution and thus have a major impact on the organization of genetic diversity within the genome. Therefore, understanding what forces play roles in shaping patterns of genetic variation, decrease in diversity, and target of selection in domesticated species is of importance to genetics of crop and animal domestication.

Population genetics analysis is a powerful approach to disentangling the effects of different evolutionary forces on DNA variation. In the past decade, there have been many efforts to investigate the patterns of genetic diversity and histories of domestication for major crops like maize (Vigouroux et al. 2002; Tenaillon et al. 2004; Wright et al. 2005; Tian et al. 2009), rice (Tang et al. 2006; Caicedo et al. 2007), soybean (Zhu et al. 2003), sunflower (Liu and Burke 2006; Olsen et al. 2006; Kolkman et al. 2007), sorghum (Hamblin et al. 2006), and tomato (Labate et al. 2009). All previous studies demonstrated that major crops have retained 30–67% of nucleotide diversity present in the corresponding wild relatives. Besides effects on overall level of diversity, domestication has led to increase in linkage disequilibrium (LD, nonrandom association of alleles at different sites) throughout a genome via population bottleneck or selection or inbreeding or changes in mating system or their combinations.

Although an increasing number of polymorphism datasets have emerged from different crop species and their wild relatives, there is little data from either domesticated or wild animals. Since animals and plants have striking differences in behavior, life cycle, environment and morphology, it is necessary to describe patterns of nucleotide diversity and extent of LD in a typical domesticated animal.

The domesticated silkworm, *Bombyx mori* L. (Lepidoptera: Bombycidae), originated from the ancient Chinese wild silkworm, *Bombyx mandarina* Moore, and has experienced a history of artificial selection for more than 5,000 years (Xiang et al. 2005). Since the beginning of last century, *B. mori* has been a model organism for the Lepidoptera order of insects because it has many morphological mutants. More than 1,000 inbred domesticated strains are maintained worldwide (Goldsmith et al. 2005). In addition, wild silkworm currently occurs in mulberry fields. *B. mori* and *B. mandarina* differ in many traits including growth rate, flight behavior, egg laying, cocoon size, silk quality, and a kind of mimicry as a result of domestication. These silkworms provide a unique opportunity for study on the evolutionary forces that have resulted in the trait differences between the two species.

Although a genome-wide resequencing of 40 silkworm genomes by Solexa has been reported recently (Xia et al. 2009), information of nucleotide diversity by the traditional sequencing method is still lacking for *B. mori* and *B. mandarina.* Thus, this study is a complementary work which may uncover some findings neglected by the resequencing, especially when pitfalls of the Solexa technique may affect the efficiency of SNP calling (Shendure et al. 2008; Pool et al. 2010). In fact, our study was launched before the silkworm Solexa resequencing project started. Nine nuclear genes were sequenced for two populations of domesticated and wild silkworms. The results suggested that domesticated silkworm lost 33–49% of nucleotide diversity relative to wild silkworms. The reduction of genetic diversity presented in this study is similar to that found in major cultivated crops (Vigouroux et al. 2002; Zhu et al. 2003;Tenaillon et al. 2004; Wright et al. 2005; Tang et al. 2006; Liu and Burke 2006; Olsen et al. 2006; Caicedo et al. 2007; Hamblin et al. 2006; Kolkman et al. 2007;Tian et al. 2009; Labate et al. 2009). We also performed coalescent simulations under a bottleneck model with bottleneck population size (*N_b_*) and duration time (*d*) to look at bottleneck intensity and detect genes departing from the model. As a result, silkworms might experience more severe bottleneck than crops. One locus, *DefA*, exhibited a departure from neutrality. Furthermore, elevated LD in domesticated silkworms is likely to be caused by bottleneck and inbreeding. The observations presented in this study are somewhat different from that of Solexa resequencing, especially in genetic diversity loss level. The possible reasons for these differences are discussed.

## Materials and Methods

### Silkworms

Sixteen domesticated silkworm strains and 15 Chinese wild silkworm samples were used in this study (Table 1). The domesticated accessions were obtained from the Institute of Sericulture and Systems Biology at Southwest University, China, and represented four main geographic strains (i.e., Chinese, Japanese, European, and Tropical) with great genetic diversity in voltine and other characters. The wild silkworm samples were collected from various geographical regions in China. Genomic DNAs were extracted from larva, pupa, or moth for each individual, using a standard phenol-chloroform approach.

### Genes studied

To investigate the general patterns of nucleotide diversity in wild and domesticated silkworms, nine loci were chosen based on their functions and locations on chromosomes (Table 2). They were all considered as neutral loci in the study using Solexa sequencing method (Xia et al. 2009). Since α-Amylase (*Amy*), Acetylcholinesterase 2 (*AchE*), and *RpSA* cannot be mapped on chromosomes in the current silkworm genome sequence, the other six loci are located on five respective chromosomes and far from each other even if on the same chromosome. In short, they were selected from at least four of 28 chromosomes and represented genes of different functions including digestion, development, immunity and reproduction. Wingless-1 (*wnt-1*) and ecdysone receptor (*ER*) are involved in development. Defensin A (*DefA*) is a member of the antibacterial peptide family and plays roles in innate immunity. Pheromone biosynthesis activating neuropeptide (*PBAN*) is thought to promote synthesis of pheromone related to reproduction. Glutathione-S-transferase (*GST*) participates in detoxification metabolism and antioxidation process. α-Amylase (*Amy*) and alcohol dehydrogenase (*Adh*) hydrolyze starch and dehydrogenate ethanol during digestion, respectively. Acetylcholinesterase 2 (*AchE*) refers to catalyzing the hydrolysis of the neurotransmitter acetylcholine at cholinergic synapses of the central nervous system, and *RpSA* is ribosomal protein SA gene.

### PCR amplification, cloning and sequencing

Primers listed in Table 2 were used to perform PCR amplification for each sample. PCR products were purified using DNA Extract Kit (Omega, www.omega.com) and then cloned into PMD19-T vector (Takara, www.takarabio.com). Clones were sequenced on both strands using BigDye chemistries (Applied Biosystem, www.appliedbiosystems.com) and Applied Biosystem 3730 DNA sequencer (Invitrogen, www.invitrogen.com) following manufacturer's protocol. Three to five clones for single individual were sequenced to exclude PCR and sequencing errors. All sequences were assembled using SeqMan in DNASTAR software version 5.01 (www.dnastar.com) and edited by Bioedit 7.01 (Hall 1999). Sequences obtained in this study have been deposited with the EMBL/GenBank Data Libraries under accession no. GQ420700–GQ420850, GQ423313–GQ423341, GQ423277–GQ423307, HM132882–HM132933.

### Statistics of diversity and recombination

The sequences were aligned by ClustalW 1.81 (Thompson et al. 1997). Insertion/deletions (indels) were excluded from the analysis. For each locus, DNAsp 5.00 (Librado and Rozas, 2009) was utilized to calculate polymorphism parameters including S (number of segregating sites), π_total_ (the mean number of nucleotide differences per site), π_a_ (average pairwise difference for nonsynonymous sites), π_s_ (average pairwise difference for synonymous sites) and θ_w_ (Watterson's estimator of 4*N_e_µ*), as well as π_silent_ (π values for synonymous and noncoding sites), θ_silent_ (θ_w_ values for synonymous and noncoding sites) and π_a_/π_s_. The minimum number (R_m_) of recombination events (Hudson and Kaplan 1985) and the estimator (R) of population recombination parameter (Hudson 1987) were also obtained by DNAsp.

### Neutrality tests

To assess neutral prediction and reveal evolutionary history between *B. mori* and *B. mandarina*, two kinds of tests were performed. Tajima's D, which was sensitive to low-frequency variants, was estimated for all sites of each locus by DnaSP. Tajima's D value was usually negative in populations suffering from selection and/or demographic events. Multilocus HKA test were performed using Jody Hey's HKA software (http://genfaculty.rutgers.edu/hey/software).

### Coalescent simulation

**Figure 1.**
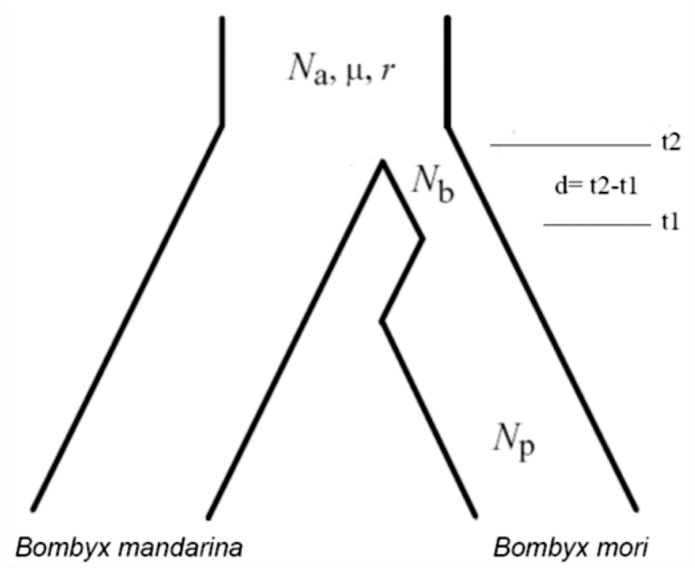
The bottleneck model of coalescent simulation in this study. High quality figures are available online.

To investigate bottleneck intensity during silkworm domestication, a single bottleneck model was introduced (Weiss and Haeseler 1998). Coalescent simulation was run using Hudson's ms (Hudson 2002) and the parameters in coalescent simulation (Figure 1) were given as follows:

-*N_a_* and *N_p_*: N_a_ was the ancestor population size before split of two silkworms and *N_p_*was the population size of present domesticated silkworm. Since previous studies have shown that there was a litter effect of population size on simulation (Wright et al. 2005; Zhu et al. 2007; Li et al. 2009), we assigned both *N_a_* and *N_p_* to 1000000.

-µ and γ: µ for each locus was estimated using θ_silent_ for wild silkworm, where µ=θ/4*N_e_*. The recombination rate (γ) for each locus was determined by γ=ρ/4*N_e_* and ρ values for wild silkworm for each locus were calculated by LDhat (http://www.stats.ox.ac.uk/∼mcvean/LDhat/instructions.html).

-*N_b_* and *d*: The key parameter of the bottleneck model was *k*, where *k* = *Nb/d. N_b_* was the population size of *B. mori* during bottleneck and *d* was the duration of bottleneck. *d* was determined by t2–t1, where t2 was the time domestication occurred and t1 was the time of the domestication termination. The archaeological evidence suggested that *B. mori* split from *B. madnarina* about 5000 years ago (Xiang et al. 2005). 5000 years should be a minimum value as the accurate time of both t2 and t1 was unknown. So we made an implicit assumption that domestication of crops and silkworm occurred at the proximate time and fixed t2 to 7500 and six *d* values (*d* = 200, 500, 1000, 1500, 2000 and 3000) were explored. The maximum *d* value was set to 3000 for two reasons. First, in coalescent simulations for crops, 3000 generation (or year) was the maximum duration of a bottleneck in crop domestication (Zhu et al. 2007). Second, 7500 minus 3000 was 4500, which was around 5000. In general, the range of *k* varied from 0.0001 to 7 and 150 combinations (scenarios) for each locus were investigated.

For each simulation at each locus, three diversity statistics were calculated, π, S (the number of segregating sites) and Tajima's D from simulated *B. mori* data. Observed values of π, S and Tajima's D from *B. mori* were compared to simulated values and fit of simulated data to observed data was assessed. To assess fit, levels of acceptance were defined corresponding to a range of ± 20% (Weiss and Haeseler 1998) of π, S or Tajima'S D. The likelihood of each scenario for each locus was obtained by calculating portion of 10000 simulations (Tenaillon et al. 2004; Wright et al. 2005; Zhu et al. 2007; Haudry et al. 2007) fitting the data. Multilocus likelihood values were calculated by multiplying across loci. This method was based on the assumption that loci were independent, which was verified by interlocus linkage disequilibrium.

### Analysis of intralocus linkage disequilibrium

Linkage disequilibrium (LD) within genes was measured as *r^2^* (squared allele frequency correlation, Hill and Robertson, 1968) between pairwise sites over physical distance. For a combined set of all nine loci, DNAsp was used to calculate *r^2^* and all singletons were excluded from LD analysis. We also evaluated distribution of *r^2^* for *DefA*, which may show selection signature. *r^2^* was plotted against pairwise distance and two logarithm curves fitting the data were drawn for *B. mori* and *B. mandarina*, respectively.

### Analysis of interlocus linkage disequilibrium

To survey extent of linkage disequilibrium between genes, interlocus LD analysis was performed across nine loci for both *B. mori* and *B. mandarina.* The number of haplotypes was measured and haplotype distribution was identified by DNAsp for each locus. Then, diploid data was generated manually to create an input file for GenePop (Raymond and Rousset, 1995). In GenePop, each haplotype was considered as an allele and significant tests of interlocus linkage disequilibrium were performed.

## Results

### Diversity

For nine loci investigated, the average sample sizes were 14.9 for domesticated silkworms and 14.7 for wild silkworms. After excluding indels, the length of alignment sequences varied from 788 to 1678 bp and a total of 9268 bp sequence per individual was sequenced, including 4368 bp of coding region and 4964 bp of noncoding region, respectively (Table 2). In contrast, the number of polymorphic sites in *B. mandarina* was nearly twice than that in *B. mori*; there were 638 polymorphic sites (1 SNP/ 14.5bp) in wild silkworm and 330 segregating sites (1 SNP/28bp) in domesticated silkworm. In Table 3, the value of *π*_total_ ranged from 0.0006 to 0.02685 with the average of 0.01166 ± 0.00912 per bp in *B. mori*, whereas it varied from 0.0052 to 0.0312 in *B. mandarina* with a mean of 0.01741 ± 0.00951 per bp. Similarly, the estimate of Watterson's *θ*_w_ fluctuated from 0.0014 to 0.01957 (mean: 0.01124 ± 0.00715) in *B. mori*, compared with that ranging from 0.0098 to 0.0326 (mean: 0.02206 ± 0.00975) in *B. mandarina.* The value of *π_total_*in *B. mandarina* was significantly higher than that in *B. mori* (Wilcoxon signed rank test, Wilcoxon 1945, *P* < 0.01, df = 8), as well as *θ*_w_ (Wilcoxon signed rank test, Wilcoxon 1945, *P* < 0.01, df = 8). *B. mori* harbors only 67% or 51% of genetic diversity relative to *B. mandarina*, measured by *π* or *θ*_w_. Non-zero estimates of π_a_/π_s_ ranged from 0.033 to 2.49 in *B. mori* and the average was 0.4 — 0.797, whereas the values of π_a_ /π_s_ ranged from 0.022 to 1.339 in *B. mandarina* and the average was 0.321 ± 0.465 (Table 3).

Synonymous and nonsynonymous sites were counted within and between species. There was only one fixed substitution between species. It is not surprising that only one nonsynonymous change occurred at *DefA*, given that domestication has only happened within the last 5,000 years (Lu et al. 2002). Such absence of fixed differences is also the case in rice (Zhu et al. 2007) and sorghum (White et al. 2004). In fact, *B. mori* and *B. mandarina* can be crossed with each other and the hybrid progeny is fertile, even though they are defined as two distinct species (Banno et al. 2004).

We also estimated π and θ_w_ for seven univoltine and five bivoltine domesticated silkworm strains, respectively. Summary statistics of nucleotide diversity did not show difference between univoltine and bivoltine strains (Wilcoxon signed rank test, Wilcoxon 1945, *P* > 0.05). Thus, all strains of *B. mori* were combined as one group in further analysis.

### Neutrality test

Tajima's D values in *B. mori* are higher than those in *B. mandarina* for eight of nine loci (Table 3), perhaps resulting from recent bottleneck in domesticated silkworms. This is consistent with the pattern observed in rice (Zhu et al. 2007) and maize (Tenaillon et al. 2004). Tajima's D values are negative at six loci for *B. mori* and at eight loci for *B. mandarina* (Table 3), suggesting that the allele frequency at these loci skewed to rare variants, which may be the result of purifying selection, subdivision or population expansion (Tenaillon et al. 2004). Three loci (*PBAN*, *AchE* and *RpSA*) in *B. mori* and one locus (*RpSA*) in *B. mandarina* showed positive D values, indicating that intermediate-frequency variants were dominant in the populations. Three loci, *DefA*, *ER* and *AchE*, exhibited significant Tajima's D values, which were likely due to either selection or demographic events.

**Figure 2.**
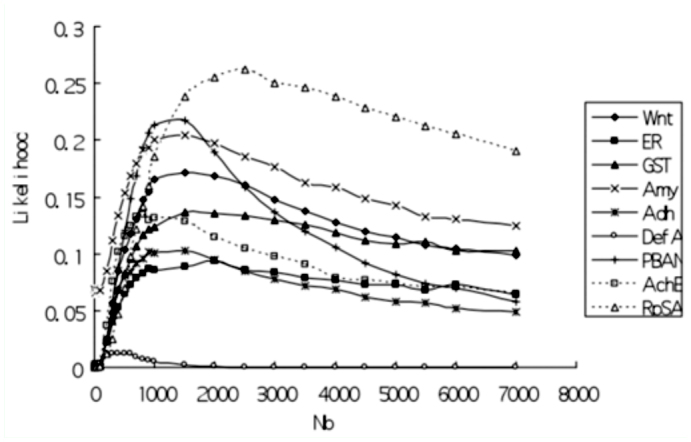
Likelihood curves of 9 loci using the number of segregating sites (S) as fit standard with d=1000. High quality figures are available online.

The ratio of π_a_ to π_s_ at *DefA* exceeded one (1.339) in *B. mandarina*, but was not available in *B. mori* because of the absence of polymorphic sites in the coding region. In fact, a sharp decrease of polymorphism at *DefA* in *B. mori* relative to *B. mandarina* measured by segregating sites was also observed in both whole sequence (4 in *B. mori* vs 85 in *B. mandarina*, Table 3) and coding region (no segregating sites in *B. mori* and 14 segregating sites in *B. mandarina*, Table 3). Thus, we can make a conclusion that *DefA* is the most likely to be a target of artificial selection in *B. mori.*

*ER*, which is subject to purifying selection because of significantly negative D values, may experience a relaxation of purifying selection based on different π_a_/π_s_ ratios for domesticated (0.832) and wild species (0.517).

A higher π_a_/π_s_ ratio (2.409) at *PBAN* in *B. mori* was observed. However, this high ratio was based on only one synonymous substitution and three nonsynonymous substitutions that occurred in the partial coding region investigated. In addition, Tajima's D value at *PBAN* in *B. mori* was not significant, suggesting that neutrality could not be ruled out.

In a multilocus HKA test, neutrality was not refused based on nine loci (data not shown). A possible reason is that *B. mori* and *B. mandarina* are closely related species and there is little divergence between the two species. The power of a multilocus HKA test is low. Thus, other analysis methods should be considered.

### Coalescent simulation and bottleneck severity

**Supplementary Figure S1:**
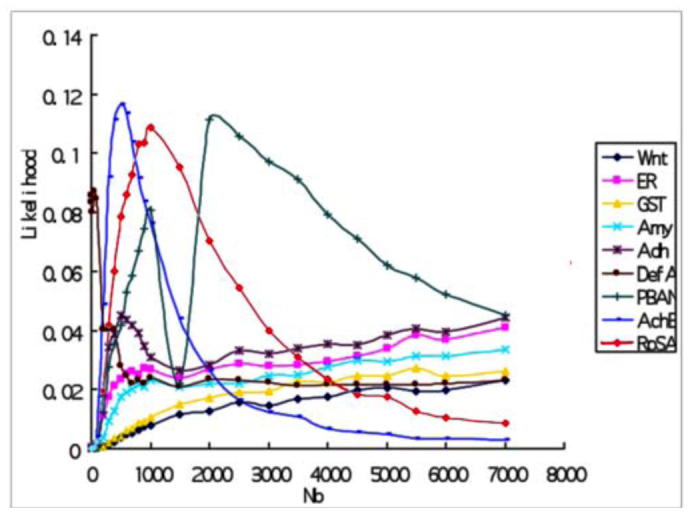
Likelihood curves of 9 loci using Tajima's D (D) as fit standard with *d* = 1000. High quality figures are available online.

Likelihood curves using *S* (the number of segregating sites) as fit standard for each locus with *d* = 1000 (*d* is the duration of bottleneck) were displayed in Figure 2. Curves of all loci showed apparent peaks except for *DefA*, suggesting that those loci fit well with one bottleneck model. Likelihood values of all loci reached peaks when *N_b_* < 2500 (*N_b_* is the population size of *B. mori* during bottleneck) (Figure 2). However, likelihood surface of *DefA* only showed a very slight peak and the likelihood values for this curve were smaller than 0.05, which is the same case at several selected loci of maize also suffering from more severe bottleneck than other neutral loci (Tenaillon et al. 2004). This suggested that *DefA* was subject to the most severe bottleneck. Thus, *DefA* deserves further analysis for selective signature. In addition, likelihood curves based on Tajima's D failed to reach peaks for 7 of 9 loci (Supplementary Figure S1). A possible reason for this is that Tajima's D skewed toward rare alleles. Distribution of Tajima's D for wild silkworms might be never mimicked adequately in simulations (Tenaillon et al. 2004).

**Figure 3.**
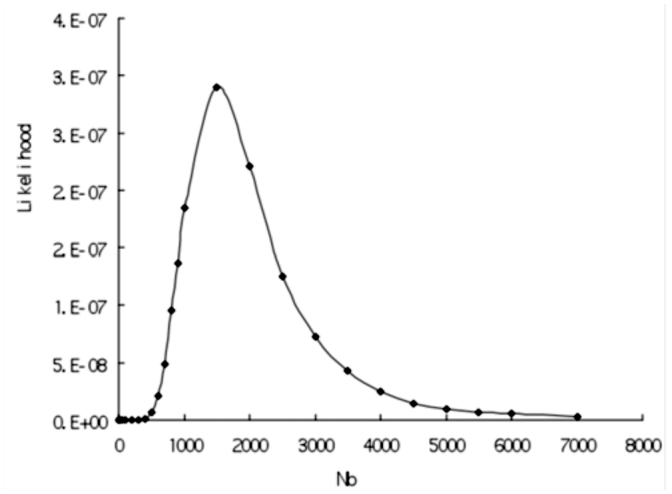
Joint likelihood surfaces of 8 neutral loci using the number of segregating sites (S) as fit standard with d=1000. High quality figures are available online.

Multilocus likelihood values were calculated to find the optimal *k* (= *Nb*/*d*) value. *DefA* was excluded from multilocus likelihood analysis because this locus showed evidence of the most severe bottleneck. Thus, multilocus likelihood curve based on eight neutral loci was obtained. Figure 3 shows multilocus likelihood surface based on *S* with *d* = 1000. Interestingly, over six *d* values, *k* values with maximum likelihood were all 1.5 using either *π* or *S* as fit- of-goodness standard. Because Tajima's D test and π_a_/π_s_ ratios suggested two loci (*ER* and *AchE*) may not evolve neutrally, we repeated multilocus likelihood analysis by removing those two loci. However, the surface changed a little (data not shown), indicating that those two loci fit bottleneck model well and may evolve neutrally.

### Test for selection using bottleneck model

To investigate whether one locus evolved neutrally, a likelihood ratio test was performed. Figure 3 shows that for eight neutral loci, multilocus likelihood reached the maximum value when *N_b_* = 1500 (*k* = 1.5) under the condition that *d* = 1000. Then, it can be obtained from Figure 2 that likelihood value for *DefA* was 0.0016 when *N_b_* = 1500 (*k* = 1.5). However, the maximum likelihood value of *DefA* was 0.0117 with *N_b_* = 300 (Figure 2). Thus, -21n(0.0016/0.0117), an likelihood ratio test statistic for *DefA* was constructed based on *S* and the condition with *d* = 1000. The test was significant (*P* = 0.045, df = 1). Similarly, the likelihood ratio test for *DefA*, using π as fit standard, was also significant (*P* = 0.017, df = 1). In addition, likelihood ratio tests for the other eight loci were not significant at all. Therefore, *DefA* did not fit the bottleneck model and was a selection target during domestication.

**Figure 4.**
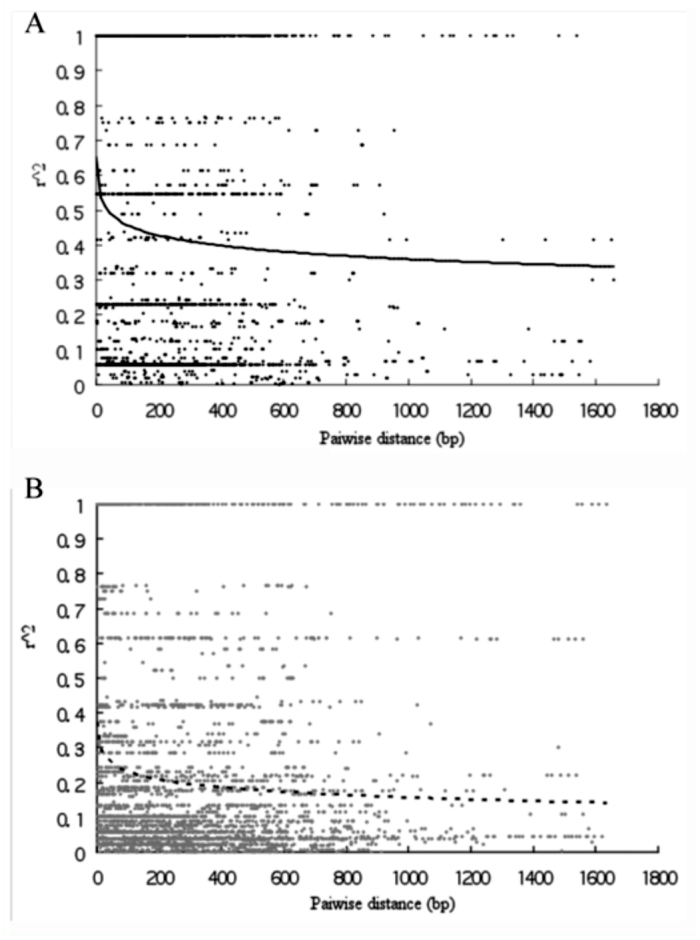
Decay of intralocus linkage disequilibrium against pairwise distance for 9 loci in domesticated silkworm (A) and wild silkworm (B). The solid and dotted lines represent logarithmic curves fitting to the data for *B. mori* and *B. mandarina*, respectively. High quality figures are available online.

### Linkage disequilibrium and recombination

Figure 4 shows the decay of linkage disequilibrium (LD) with nucleotide pairwise distance for pooled data of nine genes in each silkworm. *r^2^* refers to squared allele frequency correlation and was used to fit a logarithm curve across distances for *B. mori* and *B. mandarina*, respectively. Both curves of LD decay rapidly within 200 bp and drop slowly when distance is > 200 bp. Most strikingly, however, *B. mori* shows an extensive LD at a value more than 0.4 within 1,200 bp. In contrast, the curve for *B. mandarina* is below that of *B. mori* and drops to 0.1 within about 1600 bp.

**Figure 5.**
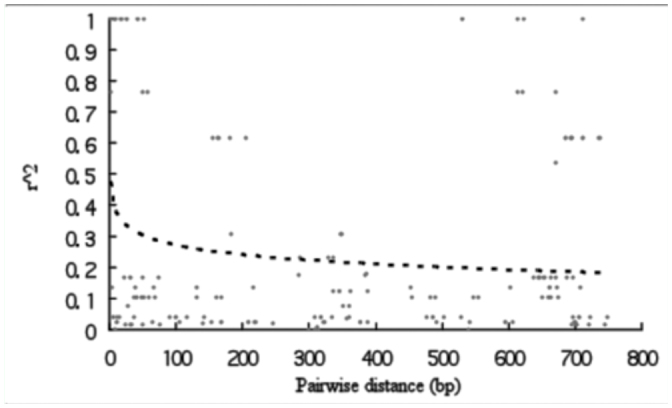
Decay of intralocus linkage disequilibrium against pairwise distance for *DefA* in wild silkworm. The dotted line represents logarithmic curve fitting to the data for *B. mandarina* only. High quality figures are available online.

We also analyzed intralocus LD for *DefA* with selection signature. However, the LD pattern at this locus was different from the one obtained for pooled data of nine genes; the curve of LD decay for *DefA* in *B. mandarina* drops to 0.2 within about 500 bp (Figure 5). However, for *DefA* in *B. mori*, there is no pairwise comparison and LD cannot be analyzed.

Estimates of recombination parameter R (Hudson 1987) range from 0 to 0.0866 (mean: 0.0183 ± 0.0288) in *B. mori* and from 0 to 0.0744 (mean: 0.033 ± 0.0262) in *B. mandarina* (Table 3). Similarly, the minimum numbers of recombination events (R_m_) range from 0 to 8 in *B. mori* (mean: 2.0 ± 2.8) and from 0 to 12 (mean: 5.6 ± 3.9) in *B. mandarina.*

**Supplementary Figure S2:**
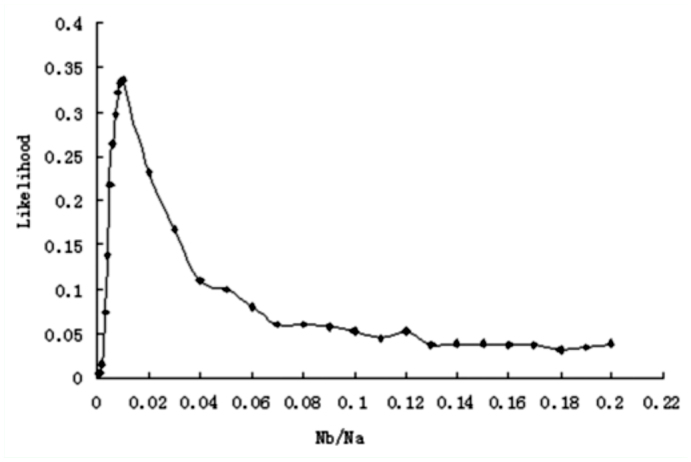
Joint likelihood curves of 8 neutral loci using S as fit standard without duration of bottleneck. High quality figures are available online.

Interlocus LD for both *B. mori* and *B. mandarina* was analyzed based on all nine loci. However, no significant results were observed in any comparisons and thus, these loci were unlinked. In fact, these loci are either on different chromosomes or on different scaffolds on the same chromosome (Table 2).

## Discussion

### Nucleotide Diversity

This study presented the nucleotide polymorphism data in domesticated and wild silkworms by traditional sequencing. Wild silkworm, which lives in natural environmental conditions, harbors substantial levels of nucleotide diversity (mean π_total_: 0.01741±0.00951, Table 3). These observations are comparable to the levels (π: 0.0018–0.0350) found in *Drosophila* (Andolfatto 2005; Matzkin 2008) and butterfly (Counterman et al. 2010). Relative to wild silkworm, domesticated silkworm contains markedly less nucleotide diversity. There was on average 33% or 49% loss of nucleotide diversity measured by π or θ calculated on nine loci in domesticated silkworm in a comparison to wild silkworm.

In contrast, a recent whole genome scan by Solexa resequencing found that domesticated silkworm lost only 17% of nucleotide diversity measured by θ relative to wild silkworm (Xia et al. 2009). However, the nucleotide diversity in *B. mori* was found to be at a very similar level measured as θ between the two studies (0.01124 vs 0.0108: this study vs Solexa). This implies that both the gene set and domesticated silkworm samples used in this study are representative although their numbers are relatively small, respectively. There is, however, the difference of nucleotide diversity in *B. mandarina* between the two studies (0.02206vs 0.0130: this study vs Solexa). Two reasons may lead to this difference. First, the whole genome scan used only 11 wild silkworm representatives from different regions (Xia et al. 2009), whereas this study used 15 wild silkworm samples from different areas in China. Nine of 15 wild samples in this study were collected from the same areas as in the research by Solexa (Xia et al. 2009). In our dataset, average θ value is 0.01878 for nine wild samples used in previous study, which is lower than that (0.02206) for 15 samples. Although the difference is not significant (Wilcoxon signed rank test, Wilcoxon 1945, *W* = 1, *P* > 0.05), different sampling strategies for *B. mandarina* may be in part responsible for the difference in θ value.

Another reason may be that Solexa resequencing underestimated the level of nucleotide diversity in wild silkworm. Sequence reads by Solexa are usually short (∼75 bp). For some mapping algorithms, sequence reads with more than one or two differences from a reference genome will not be placed (Li et al. 2008). As Pool et al. (2010) pointed out, this makes the mapping of alleles that are different from the reference genome less probable than for a reference-matching allele, causing a bias in allele frequency towards the allele found in the reference sequence. As a result, it may underestimate level of nucleotide diversity for divergent sequences. Since one reference genome sequence was available only for *B. mori* and used for calling SNPs in both *B. mori* and *B. mandarina*, underestimation of nucleotide diversity in *B. mandarina* can be envisioned. Thus, the available reference genome sequence of *B. mandarina* will be a key factor to correct this bias in the future.

In principle, domestication leads to loss of genetic diversity in domesticated species. Interestingly, the level of nucleotide diversity loss observed in this study is comparable to the levels observed in major cultivated crops for which loss of genetic diversity is in general 30% – 69% (Table 4). It appears that domestication has a similar quantitative effect on reduction of genetic diversity in diverse domesticated species including plants and animals.

### Bottleneck intensity

In our study, *k* values with different domestication durations were all 1.5, contrasting to those in main crops that vary from 2.45 to 5 (Tenaillon et al. 2004; Wright et al. 2005; Haudry et al. 2007; Li et al. 2009). Although a previous study reported *k* = 0.8 for Asian rice (Zhu et al. 2007), probably this small *k* value may be due to high selection pressure on functional regions (Li et al. 2009). Thus, we can conclude that domesticated silkworms experienced more severe bottleneck during domestication than crops. These results indicated that the foundation population size varied from 300 (*d* = 200) to 4500 (*d* = 3000). Given duration of domestication as 1000, the foundation population size was 1500 (Figure 3) for *B. mori.* Although *B. mori* might suffer from more severe bottleneck, the reduction of nucleotide diversity was 33% or 49% relative to *B. mandarina*, a decrease that still falls into the range of diversity loss in crop domestication (Table 4). It is most likely that changes in mating systems may play an important role and contribute to loss of genetic diversity in plant domestication. The transition from open-pollinated to self-fertilization might have led to a decrease in effective population size and recombination rate (Charlesworth et al. 2003). As animals cannot self-fertilize, *B. mori* should maintain a relatively high level of polymorphism than selfing crops. As expected, we observed that the levels of nucleotide diversity (π_total_: 0.01166±0.00912, Table 3) in domesticated silkworms are somewhat higher than those found in some selfing crop plants. For example, nucleotide diversities (π_Total_) of selfing crops are 0.0023 in rice (Caicedo et al. 2007), 0.0022 in sorghum (Hamblin et al. 2006) and 0.0013 in soybean (Zhu et al. 2003). Thus, change of mating system may be a factor in determining levels of genetic diversity in crops. Without such a change, *B. mori* maintains relatively higher genetic diversity than crops.

Although a bottleneck model for silkworm domestication was also constructed recently (Xia et al. 2009), it should be pointed out that the bottleneck model neglected duration of bottleneck and, as a result, *k* value was not estimated in the previous study (Xia et al. 2009). Given *k* value with 1.5 for different *d* values, we can infer foundation population size of silkworm and compare it to other domesticated species. We also performed coalescent simulations using a bottleneck model without duration. Based on eight neutral loci, *B. mori* lost 99% population size relative to its ancestor (Supplementary Figure S2) compared with 90% reduction of population size in previous study (Xia et al. 2009). Both decreases in population size of *B*. *mori* suggest a severe bottleneck during domestication.

### Target of selection

One of the goals of this study was to test selection under the bottleneck model. Among nine loci, only *DefA* showed evidence of selection. As one of the factors involved in the innate immune system, *DefA* was involved in resistance to microbes. Strikingly, no polymorphism was observed in *DefA* coding sequences of the domesticated silkworm whereas a normal level of nucleotide diversity in the corresponding sequences was found in the wild silkworm (Table 3). These results can be explained by the fact that *DefA* has experienced a strong artificial selection in the domesticated silkworm. It is most likely that during domestication, human beings selected strongly healthy individuals to raise and breed. As a result, polymorphism at the locus decreased gradually and finally one haplotype with the best fitness was fixed in domesticated silkworm. In contrast, *B. mandarina* was subject to purifying selection rather than such strong artificial selection and thus contained a normal level of polymorphism (π: 0.0202) at *DefA.* Thus, *DefA* may be a target of selection during domestication.

In recent research, a large number of genes involved in immunity, including many unique recognition genes and antimicrobial peptide genes in domesticated silkworm, were identified (Tanaka et al. 2008). Thus, it is interesting to investigate nucleotide diversity at antimicrobial peptide gene loci in silkworms to see whether innate immune systems will be really critical for silkworm domestication in the future.

Although *ER* and *AchE* also showed significant Tajima's D value and *PBAN* harbored high π_a_/π_s_ ratio, neither of them departed from neutrality in a coalescent simulation test. In fact, it is disingenuous to test neutrality just using Tajima's D test because significance of Tajima's D can result from factors including selection and/or demographic event. Similarly, π_a_/π_s_ ratio based on a small segment of coding sequence was weak in the neutrality test. In our study, these three loci fit well with a bottleneck model and thus, they were considered as neutral genes. In contrast, departure from neutrality for *DefA* has been proven by Tajima's D test, π_a_/π_s_ ratio and coalescent simulation. Therefore, selection detection by coalescent simulation is the most reliable, which is also the case in maize (Tenaillon et al. 2004; Wright et al. 2005).

### Linkage disequilibrium

In Figure 4, increase in the extent of LD observed in *B. mori* was observed, corresponding to a small mean number of R_m_ (2.0 ± 2.8) and low recombination parameter (0.0183 ± 0.0288). Reduction of effective population size and/or artificial selection on domestication loci may be responsible for this. It is notable that in both silkworm and crops, Tajiama's D values were higher in domesticated species than those in wild ones (see Results section). Thus, recent bottleneck or inbreeding could not be ignored. However, we cannot distinguish bottleneck and inbreeding in the case of absence of domesticated silkworm landraces. In the future, it would be wise to sequence the genes in silkworm landraces in order to investigate corresponding diversity and LD patterns.

Compared with Figure 4A, *DefA* showed absence of decay of LD in *B. mori.* In fact, *B. mori* was extremely homozygous at *DefA* (Table 3). Thus, it can concluded that selection resulted in lower recombination at this locus and should be responsible for extensive LD in *B. mori.*

As in an outcrosser, *r*^2^ declines to 0.10 and 0.4 within 1.6 kbp in wild silkworm and domesticated silkworm, respectively. Similarly, *r*^2^ declines to < 0.10 within 1.0 kbp in maize, a crop that is highly outcrossed (Tenaillon et al. 2001; Remington et al. 2001). However, selfing crops showed somewhat different patterns in LD decay. For example, there is little decline in LD over distances as great as 50 kbp in soybean (Zhu et al. 2003); LD in rice approaches *r*^2^ = 0.10 only after about 100 kbp (Garris et al. 2003). Thus, LD patterns of both silkworms in this study are similar to those of outcrossing crops.

### Conclusions

Diversity of *B. mori* was significantly lower than that of *B. mandarina* measured as π_total_ (0.01166 vs. 0.1741) or *θ*w (0.01124 vs. 0.02206). In general, *B. mori* lost 33–49% of nucleotide diversity relative to wild silkworm and the reduction of genetic diversity is similar to the levels found in major cultivated crops. *B. mori* suffered from more severe bottleneck than crops. *DefA* showed evidence of selection using either a neutrality test or coalescent simulation. As a kind of immunity-related gene, *DefA* perhaps experienced strongly artificial selection and became highly conserved in *B. mori* during domestication. Elevated LD in *B. mori* was observed and may be an indicator of selection and demographic events. LD patterns of both silkworms in this study are similar to those of outcrossing crops.

**Table 1.**
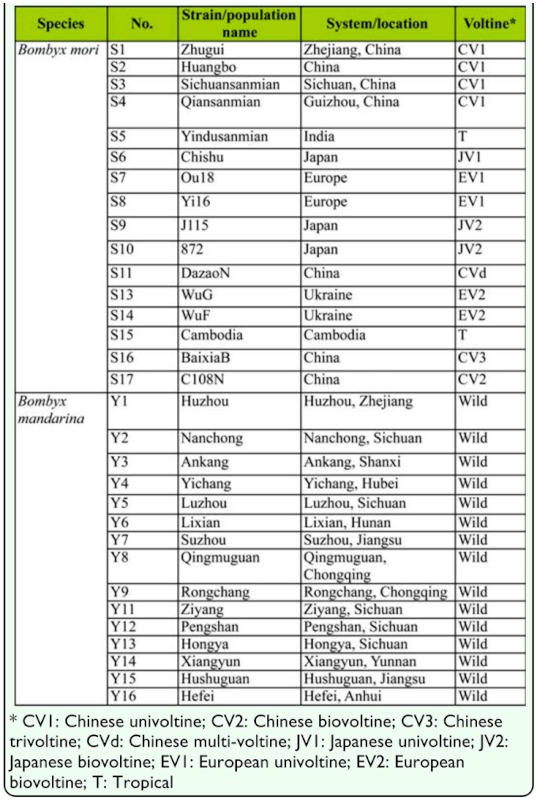
Characters and source of materials used in this study

**Table 2.**
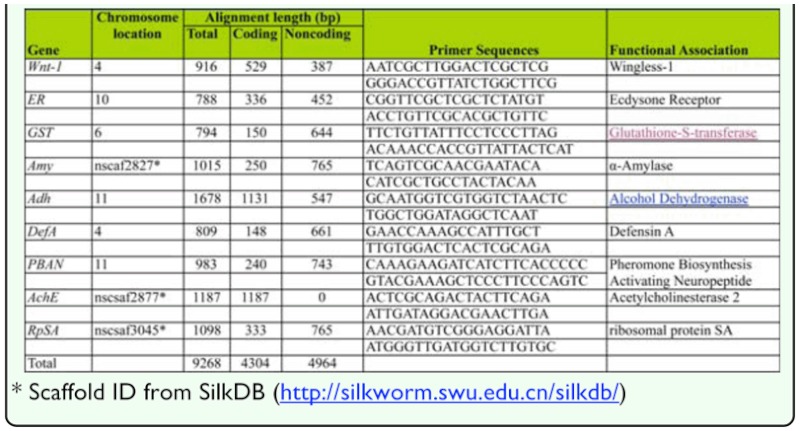
Summary of genes surveyed and primer sequences employed in this study.

**Table 3.**
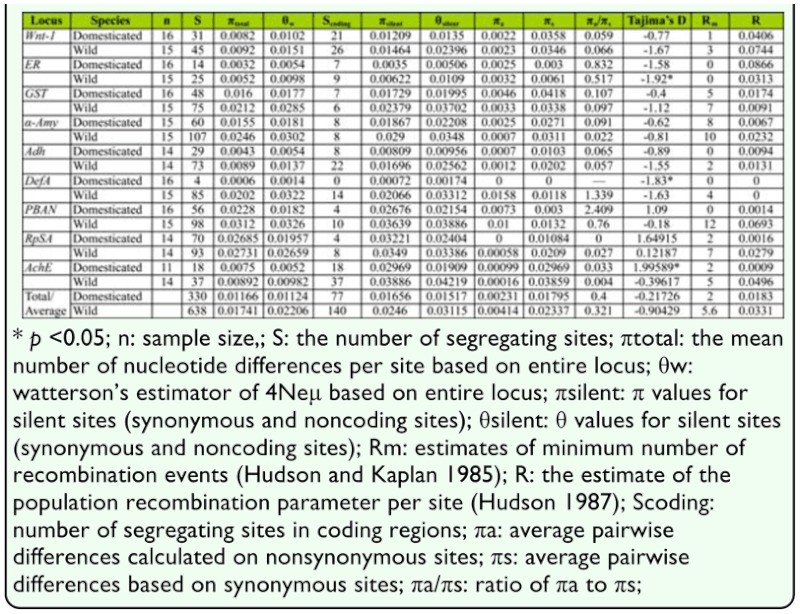
Summary of diversity in *B. mori* and *B. mandarina.*

**Table 4.**
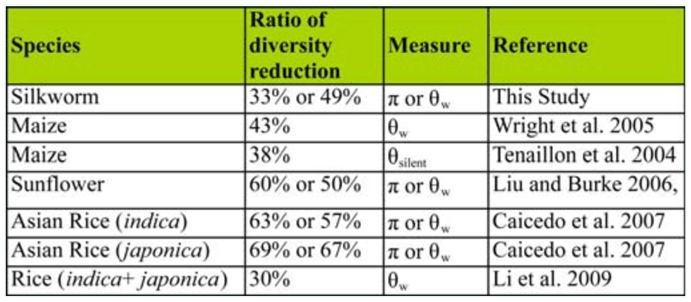
Reduction of diversity in domesticated species relative to their wild species.

## Abbreviations

**LD**,: linkage disequilibrium
